# An evidence-based evaluation of transferrable skills and job satisfaction for science PhDs

**DOI:** 10.1371/journal.pone.0185023

**Published:** 2017-09-20

**Authors:** Melanie Sinche, Rebekah L. Layton, Patrick D. Brandt, Anna B. O’Connell, Joshua D. Hall, Ashalla M. Freeman, Jessica R. Harrell, Jeanette Gowen Cook, Patrick J. Brennwald

**Affiliations:** 1 The Jackson Laboratory for Genomic Medicine, Farmington, Connecticut, United States of America; 2 Office of Graduate Education, University of North Carolina at Chapel Hill, Chapel Hill, North Carolina, United States of America; 3 Department Biochemistry & Biophysics, University of North Carolina at Chapel Hill, Chapel Hill, North Carolina, United States of America; 4 Department of Cell Biology & Physiology, University of North Carolina at Chapel Hill, Chapel Hill, North Carolina, United States of America; Utrecht University, NETHERLANDS

## Abstract

PhD recipients acquire discipline-specific knowledge and a range of relevant skills during their training in the life sciences, physical sciences, computational sciences, social sciences, and engineering. Empirically testing the applicability of these skills to various careers held by graduates will help assess the value of current training models. This report details results of an Internet survey of science PhDs (*n* = 8099) who provided ratings for fifteen transferrable skills. Indeed, analyses indicated that doctoral training develops these transferrable skills, crucial to success in a wide range of careers including research-intensive (RI) and non-research-intensive (NRI) careers. Notably, the vast majority of skills were transferrable across both RI and NRI careers, with the exception of three skills that favored RI careers (creativity/innovative thinking, career planning and awareness skills, and ability to work with people outside the organization) and three skills that favored NRI careers (time management, ability to learn quickly, ability to manage a project). High overall rankings suggested that graduate training imparted transferrable skills broadly. Nonetheless, we identified gaps between career skills needed and skills developed in PhD training that suggest potential areas for improvement in graduate training. Therefore, we suggest that a two-pronged approach is crucial to maximizing existing career opportunities for PhDs and developing a career-conscious training model: 1) encouraging trainees to recognize their existing individual skill sets, and 2) increasing resources and programmatic interventions at the institutional level to address skill gaps. Lastly, comparison of job satisfaction ratings between PhD-trained employees in both career categories indicated that those in NRI career paths were just as satisfied in their work as their RI counterparts. We conclude that PhD training prepares graduates for a broad range of satisfying careers, potentially more than trainees and program leaders currently appreciate.

## Introduction

The original academic training model was designed as an apprentice model for a single career pathway. In that model, the faculty advisor served as a mentor, guiding each trainee towards a future position as a tenure track faculty member. Old assumptions regarding the natural progression of PhD scientists into faculty careers are rapidly changing to reflect a job market where only a small percentage of PhDs will follow in their academic advisors’ footsteps (e.g., [1, 2, 3, 4]). Over time, the number of PhDs conferred in scientific disciplines in the U.S. [3] has risen substantially, increasing the pool of applicants for tenure-track faculty positions [5]. Hence, career progression for today’s PhD graduates is quite different. Instead of resembling a continuous pipeline, training outcomes have been compared to a *branching* career pipeline [1, 6].

Because of the traditional structure of the academy, an outdated perception still persists that the best academic trainees in the sciences pursue faculty careers [7], and that the traditional tenure-track career path represents the true path to job satisfaction. Furthermore, a perception persists that doctoral trainees in the sciences and related fields develop discipline-specific skills that are relevant only to field-specific research, limiting options for other career choices. This study aimed to test these assumptions. We have chosen to focus broadly on disciplines which place an emphasis on employing the scientific method to engage in research, including life sciences, physical sciences, computational sciences, social sciences, and engineering disciplines. Social sciences are included because of their common reliance on the scientific method for research and training. Sometimes these disciplines are collectively referred to as “STEM” (science, technology, engineering, and mathematics); while there is merit to considering some of these academic areas separately [8, 9], this broad focus is consistent with *Science and Engineering Indicators* published by the National Science Foundation which includes all of the disciplines outlined herein [10]. For the purposes of this study, we will refer to this group collectively as “science PhDs.” First, we identify skills developed by these PhD-level trainees. Next, we highlight similarities and/or differences in the development of transferrable skills for those who pursue research-intensive (RI) versus non-research-intensive (NRI) careers. Finally, we explore potential job satisfaction differences between the two broad career groups.

Many graduate institutions struggle to adjust to new job market realities, continuing to provide training primarily for tenure-track faculty positions. However, there are an increasing number and range of opportunities for PhD-trained scientists that lead to distinguished careers in both RI and NRI career pathways (i.e., [11, 12, 13]). This evolution of the career landscape challenges the apprentice model for doctoral training wherein PhDs are prepared primarily for faculty research positions instead of the wide variety of jobs available to them.

Despite recent reports of doctoral program alumni entering NRI careers [2, 14, 15], empirical data related to transferrable skill development and associated career outcomes are lacking. Trainees have limited familiarity with rapidly evolving hiring trends and the wide range of job opportunities available; this could be remedied by greater visibility of PhD career outcomes data [16]. Previous research has focused on faculty positions (e.g., comparing the number of faculty positions available to graduate school enrollment numbers), or is limited to specific disciplines rather than cross-disciplinary samples (e.g., biomedical fields) [15, 17]. We have therefore engaged in an extensive study of skills developed during PhD training across scientific disciplines, and of the contribution of these skills to career outcomes and job satisfaction for PhDs employed in a variety of careers. The results of this analysis can inform efforts to improve professional development in doctoral training programs. The current analysis begins to address this knowledge gap by: examining skill development during training relative to skill importance in actual positions obtained, and examining job satisfaction for PhDs in their post-training employment role.

The purpose of this study is to identify skills important to the success of both major career trajectories (RI and NRI) and to determine whether transferrable skills for both were developed during PhD training. We define RI careers as those careers where the scientist is conducting research as a primary function of the job. NRI careers include research-related careers, which require scientific knowledge but are not directly related to conducting research (see Materials and methods for listed example careers in each category). Our sample includes respondents who indicated they were in the following disciplines: life sciences, physical sciences, computational sciences, social sciences, and engineering, allowing us to identify a core set of skills developed in scientifically-based doctoral training. The analysis draws on recently-collected survey data to determine skill attainment for those entering both RI and NRI careers and also examines job satisfaction for each career group.

We hypothesized that many skills acquired during doctoral training correspond with the needs of employers in both RI and NRI careers, yet we anticipated that development of certain skills may be preferentially beneficial to certain career fields. We further hypothesized that some skills important for career success are inadequately developed through doctoral training, and we term these “skill gaps.” To identify these classes of competencies, we examined the development of transferrable skills during doctoral training and compared ratings of self-assessed skill development with ratings of the importance of those same skills for job performance in subsequent employment. In this manner, we measured and tested how effectively graduate training prepares PhDs for a wide range of careers. Furthermore, we evaluated whether there were any differences between RI and NRI careers in job satisfaction. Finally, we discuss the implications of our findings for graduate training in the sciences and provide specific recommendations that address development of skills that are especially important in all careers available to PhDs.

## Materials and methods

### Procedures

#### Ethics statement

In compliance with ethical standards of research, all research was conducted under the auspices of the Harvard University Committee on the Use of Human Subjects, IRB#15–063. All participants were provided with an opportunity to review and agree to an informed consent as part of the online survey. All data is reported in aggregate or with identifiable information removed [7].

#### Data collection

The sample includes graduates who earned a PhD in life, physical, computational, and social sciences or engineering, at any institution between 2004–2014, and who had worked, trained, or studied in the U.S.

The sample was developed using qualitative and quantitative methods of data collection and analysis. An online survey was constructed first as a pilot, with embedded cognitive questions. The survey was then pilot tested with a small sample of respondents, revised, and a large-scale survey was launched. This large-scale data collection effort took place in the spring of 2015.

Outreach to build the sample was conducted electronically via social media channels and direct emailing to potential respondents. The total number of complete responses to the final online survey was 8,099. The current analyses include a subset of relevant questions and responses. For more detailed information on sampling techniques and survey construction, see [7].

### Participants

#### Demographics

Eighty-three percent of respondents were US Citizens or permanent residents; seventeen percent were not, and less than one percent indicated that they preferred not to respond. Seventy-seven percent identified as Caucasian, followed by thirteen percent Asian American. Underrepresented minority respondents included four percent Hispanic/Latino, two percent Black/African American, two percent other, and less than one percent Native Hawaiian/Pacific Islander or American Indian/Alaska Native (the remaining three percent preferred not to respond). Females made up fifty-seven percent of the sample; males forty-three percent; transgender, other, or those indicating a preference not to respond comprised less than one-percent.

### Measures

The final survey instrument took approximately 15 minutes to complete and included questions about career interests, activities, current employment, and motivations for career choices, and job satisfaction ratings (for additional information see [7]). The instrument consisted of four major sections: Education, Postdoctoral Training, Employment, and Demographics. The current research included a subset of these data as detailed in the measures section (see S1 File).

#### Education, training, & employment

In the Education section, participants were asked to identify the field/discipline/academic program of their PhD by selecting from a list of academic program options (including “other” write in; see S2 File). The list of doctoral programs was generated (requested by MS) using the publicly available database of the Integrated Postsecondary Education Data System (IPEDS), a division of the National Center for Education Statistics, and includes disciplines in the life sciences, physical sciences, computational sciences, social sciences, and engineering. Participants indicated whether they had completed or were currently in a postdoctoral position. Those who had postdoctoral training experience were asked to indicate the total number of years of postdoctoral training across all locations and research institutes, from 0–29 years (intervals of 1, up to 29; or 30 and above; for the purpose of analyses, this variable approximated an interval variable and was treated as such). Respondents were asked to select their current job title from a multiple-choice list (including “other” and “I don’t know”; see S3 File for full list of options), and to identify their current employer using an open-response text box. For all list options (e.g., doctoral discipline, job title) an open-response “other” text box option was provided for any answers not included on the list.

#### Academic programs represented

Respondents represented over 500 doctoral academic programs in scientifically-based fields. Selections were made from an extensive drop down list of 485 academic program options, and an additional 56 “other” options were identified in the open response section (see S2 File for full list). Respondents were grouped into five categories to examine the representativeness of the sample. All five of these programmatic areas were represented (see Table 1). A representative sample drawn from the 2015 iteration of the National Science Foundation’s Survey of Earned Doctorates suggests that the numbers of life, physical, and computational scientists in this sample are representative of the national breakdown, although the numbers of social scientists and engineers in this sample is quite low [18]. Nonetheless, a sizeable number from each group are represented.

**Table 1:** Trainee status by academic program.

Work Status	Training Status	Life Sciences	Physical Sciences	Social Sciences	Engineer-ing	Compu-tational Sciences	Sub Totals	
*Current Postdoc*	*Postdoc Still In Training*	2172	475	191	237	126	*3201*	*45%*
*Employed Sample*	*Employed*,*Postdoc Complete*	1469	492	172	143	90	*2366*	*33%*
	*Employed*, *Without Postdoc*	711	320	217	170	135	*1553*	*22%*
*Sub Totals*		*4352*	*1287*	*580*	*550*	*351*	*7120*	*100%*
		61%	18%	8%	*8%*	*5%*		

#### Job categories represented

Respondents selected the job title category most applicable to them from multiple-choice selections provided. Each multiple choice job title was later categorized as either RI or NRI, and further subdivided into one of thirteen general job categories (recoded using syntax in the IBM SPSS statistics/data management program). The four RI categories included tenure track, non-tenure track academic, government, or industry research. The eight research-related NRI categories included: administrative, business development, consulting, intellectual property, regulatory affairs, science writing and communication, teaching intensive, or science policy. Respondents who indicated that their primary job duty focused on teaching (education at a liberal arts college, community college, or K-12 institution) were included in an NRI “teaching-intensive” category, rather than in the RI category, although they may also conduct research in some cases. The remaining category consisted of “other” entries, which were binned manually into the pre-selected multiple-choice categories where applicable (of the 635 free-response text answers, 481 were sorted into existing categories, leaving 154 “other” job titles remaining). A bivariate proxy variable was created for RI versus NRI (including the research-related careers plus other responses) for use as the dependent variable for the binary logistic regression. The distribution of respondents in each of the thirteen career categories is included in Table 2.

**Table 2:** Frequency count by primary employment categories (n = 3803).

Career	Employment Category	*Employees*	*Percentage*
***Research-Intensive***	Tenure Track Research	655	17%
	Industry Research	621	16%
	Non-Tenure Track Academic Research	410	11%
	Government Research	339	9%
	**Research-Intensive Subtotal**	**(2025)**	**(53%)**
***Non-Research-Intensive***	Teaching Intensive Careers	640	17%
	Administrative	487	13%
	Consulting	168	4%
	Other	142	4%
	Business Development	97	3%
	Science Writing and Communication	92	2%
	Science Policy	69	2%
	Intellectual Property	55	1%
	Regulatory Affairs	28	<1%
	**Non-Research-Intensive Subtotal**	**(1778)**	**(47%)**
**Total**		**3803**	**100%**

“Other” respondents who indicated trainee status in text were excluded (n = 18, <1%). Only respondents currently employed outside of a postdoc are included in the table above. The employment categories above reflect a little over half of the sample; remaining respondents not included in this table are: currently employed postdocs (see Table 1), and unemployed respondents (n = 185, <3% of total respondents).

#### Transferrable skills

Skill-based questions consisted of fifteen specific transferrable skills, developed from previous work by the National Postdoctoral Association and the Career Success Program at Michigan State [7]. Each of the fifteen transferrable skill items developed during doctoral training were rated by asking respondents to indicate their level of agreement with the following statement: “I developed/continue to develop this skill during my doctoral program.” Respondents rated each skill on a 5-point Likert-type scale (1 = lowest, 5 = highest) from “strongly disagree” to “strongly agree.” A comparable rating scale was used to evaluate each skill’s importance for success in each respondent’s current position, using the following statement: “Which skills are important for success in your current position?” Respondents rated each skill on a 5-point, Likert-type scale (1 = lowest, 5 = highest) from “not at all important” to “extremely important.” Examples of transferrable skills included items like communication skills, time management, teamwork, decision-making, creativity/innovation, etc.

#### Job satisfaction

One item assessed this construct, namely “How satisfied are you in your current position?” Respondents indicated their job satisfaction on a 5-point scale (1 = lowest, 5 = highest) from “very dissatisfied” to “very satisfied.”

## Results

### Transferrable skills: Development & importance

To evaluate skill acquisition during graduate school, we first asked respondents to rate their own development of the fifteen transferrable skills during doctoral training on a five-point scale (a value of five corresponded with “strongly agree”). All means were above neutral (e.g., greater than 3.0), indicating that for nearly all of the transferrable skills, trainees generally agreed that they had developed the skill during doctoral training (Table 3). Notably, the means for the majority of skills developed during doctoral training were higher than neutral (e.g., closer to or above 4.0, indicating agreement), suggesting that PhDs across scientific disciplines do indeed build essential skills beyond discipline-specific knowledge.

**Table 3:** Means of transferrable skills during training and employment.

*Transferrable Skills*	*Doc Skill Mean*	*Skill Gap*	*Employed Skill Mean*
Discipline-specific knowledge	4.73	0.57	4.16
Ability to gather and interpret information	4.69	0.13	4.56
Ability to analyze data	4.66	0.34	4.32
Oral communication skills	4.38	-0.21	4.59
Ability to make decisions and solve problems	4.37	-0.22	4.59
Written communication skills	4.36	-0.17	4.53
Ability to learn quickly	4.18	-0.26	4.44
Ability to manage a project	4.18	-0.24	4.42
Creativity/innovative thinking	4.12	-0.11	4.23
Ability to set a vision and goals	3.99	-0.25	4.24
**Time management** §	**3.87**	-0.73	**4.60**
**Ability to work on a team** §	**3.66**	-0.77	**4.43**
**Ability to work with people outside the organization** §	**3.46**	-0.80	**4.26**
**Ability to manage others** §	**3.26**	-0.73	**3.99**
**Career planning and awareness skills** §	**3.05**	-0.52	**3.57**

Mean ratings for skills developed during doctoral training (Doc Skill Mean) and for skill importance for success on the job (Employed Skill Mean) as rated on a 5-point scale (1 = lowest, 5 = highest). Standard deviations for each skill ranged from 0.73–1.22 for doctoral skill ratings, and from 0.67–1.14 for employment skill ratings. A skill gap denotes the differences between on-the-job skill importance ratings and doctoral skill development ratings, where negative numbers indicate higher importance rating of a skill compared with the skill development rating. Adapted with permission [7].

^§^ Indicates identified skill gap

We then asked respondents to rate the importance of each skill for their current job (rated on a five-point scale; a value of five corresponded with “extremely important”). A skill gap was identified as the average difference between how well a given skill was acquired during doctoral training and the corresponding rating of the importance of that skill during employment (see Table 3). No skill gaps were greater than or equal to one point, suggesting that skill acquisition during doctoral training was generally commensurate with job requirements post-PhD training. If the importance of a skill for a particular job was rated higher than the development of that skill during doctoral training, this is indicated by a negative number. Consequently, if a particular skill was developed to a greater extent during graduate training than its perceived importance to a particular job, this is indicated by a positive number. Hence, a positive value indicates that the skill was highly developed during training and/or may not have been as necessary during employment. A negative value of larger magnitude (e.g., gaps of magnitude greater than or equal to -0.5 which would round to a deficit of -1) we identified as indicating potential areas for improvement in graduate training programs.

In addition to trends noted in the means displayed in Table 3, additional analyses produced the same trends when examining the skill gaps within subjects. Trainees were well prepared during doctoral training for the top three skills (mean differences ranged from 0.13 to 0.58; Table 3), whereas they were not as well prepared for the remainder of the skills (mean differences ranged from -0.12 to -0.89). Paired-sample t-tests for all skills were statistically significant at *p* < 0.05, *df* = 3718 to 3800, *t*s = 30.16 to -41.20. Yet based on our minimum criteria of difference (a skill gap of greater than or equal to one point), we identified five skills as those showing a substantial differences of interest. Concurring with our descriptive data, the five skills that showed both significant *and* substantial differences were the same five skills identified with the lowest developmental ratings, replicating the pattern visible in Table 3.

### Transferrable skills between career groups: RI and NRI skill importance & development

**Fig 1:**
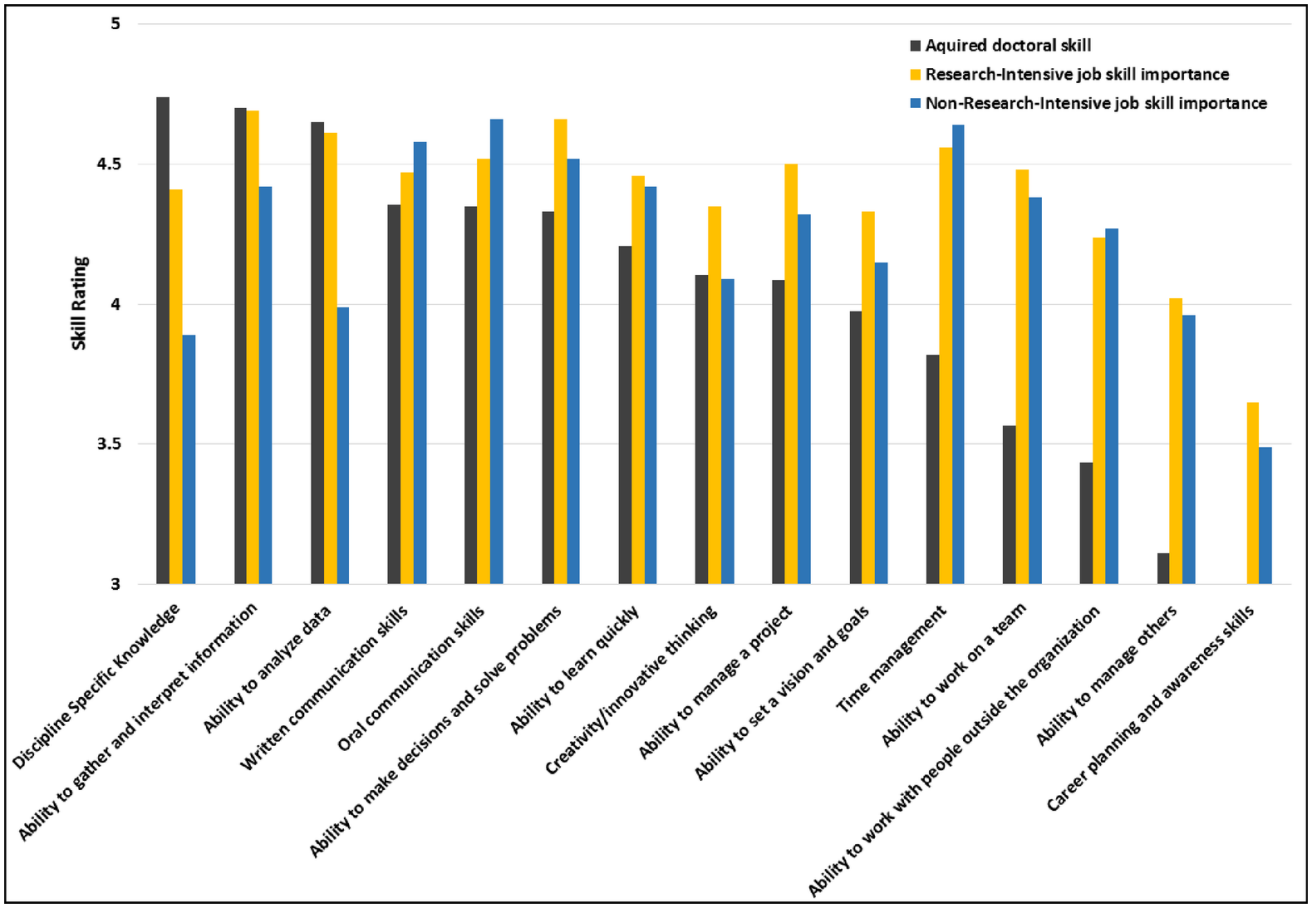
Transferrable skills: Acquired doctoral skills and skill importance ratings in research-intensive and non-research-intensive careers (means). Fig 1 Skills ordered from left (highest) to right (lowest) using transferrable skill ratings acquired during doctoral training as the reference category.

To further examine the differences between doctoral skill training and employment skill importance relative to career groups, both RI and NRI employment skills were compared with doctoral skill training (Fig 1). Fig 1 illustrates that generally the skill importance ratings were consistent between career categories. As in Table 3, which indicated only small skill gaps for the majority of skills, Fig 1 illustrates that skill development and importance means were quite similar. We continued our investigation by examining the data using further analyses to determine whether specific skills were particularly well developed for these two groups (RI and NRI; Table 4).

**Table 4:** Logistic regression of transferrable skills acquired during doctoral training on research-intensive or non-research-intensive career, controlling for postdoctoral training.

*Variables*	*Odds Ratio*	*95% CI*
*Control*		
Postdoctoral Experience** **(RI)**	**1.23**	**1.19–1.27**
*Transferrable Skills—Research-Intensive*		
Creativity/innovative thinking** **(RI)**	**1.36**	**1.23–1.51**
Career planning and awareness skills** **(RI)**	**1.19**	**1.11–1.27**
Ability to work with people outside the organization** **(RI)**	**1.13**	**1.05–1.21**
*Transferrable Skills—equally important*		
Discipline-specific knowledge	1.07	0.91–1.27
Ability to analyze data	1.05	0.89–1.24
Oral communication skills	1.00	0.89–1.14
Ability to make decisions and solve problems	1.03	0.90–1.17
Ability to set a vision and goals	1.02	0.92–1.14
Written communication skills	1.01	0.89–1.14
Ability to work on a team	0.96	0.88–1.05
Ability to manage others	0.94	0.87–1.02
Ability to gather and interpret information	0.89	0.73–1.10
*Transferrable Skills—Non-Research-Intensive*		
Time management* **(NRI)**	**0.91**	**0.84–0.99**
Ability to learn quickly* **(NRI)**	**0.88**	**0.80–0.98**
Ability to manage a project** **(NRI)**	**0.79**	**0.72–0.87**

Transferrable skills developed during doctoral training were used to predict career category using a logistic regression. Skills that differ by career category have significant p-values, indicated by asterisks and bold lettering. Transferrable skills presented in order of odds ratio magnitude, from largest (>1.0 favoring RI) to smallest (<1.0 favoring NRI); values of approximately 1.0 (with confidence intervals that include 1.0) suggest no difference between the two categories.

* Indicates p < .05

** Indicates p < .001

#### Logistic regression model

We used a logistic regression model to determine whether particular transferrable skills were associated preferentially with one of the two major career categories (e.g., RI or NRI). Logistic regression is a statistical procedure that allows for comparison of the odds of two events, in which 1.0 means that the odds of either event are equally likely, using a model of parameters entered (e.g., factors researchers believe impact the outcomes; [19]). While a few skills associated specifically with one career category versus the other (three each for RI and NRI), the majority of the skills were not associated more with either RI or NRI careers, indicating their wide applicability. To control for possible effects of postdoctoral training on skill development, we included years of postdoctoral experience as a control variable. (There was a significant positive association with length of postdoctoral experience and RI careers).

The level of association between skill acquired during training and subsequent career choice is presented in rank order from most likely to be in RI positions, to most likely to be in NRI positions (Table 4). Skills related to an RI career choice are indicated by significant odds ratios greater than one, whereas skills related to an NRI career are indicated by significant odds ratios less than one. Non-significant odds ratios (the majority of odds ratios are in the middle, close to 1.0) indicate approximately equivalent relevance for either career category. Further, the logistic regression model provided a good fit for the data, χ^2^ (16, *N* = 3579) = 268.48, p < .001, Cox & Snell *r*^2^ = .07, Naglekerke *r*^2^ = .10.

Nine of the fifteen skills developed in training showed no differential association with career category, suggesting the value of most skills irrespective of career category. However, three skills were specifically associated with one or the other career category (RI vs. NRI). Creativity/innovation, career planning/awareness, and ability to work with others outside the organization were positively associated with RI careers. In other words, respondents who rated themselves as proficient in these skills were more likely to be employed in research intensive careers. On the other hand, respondents who had high ratings for project management, learning quickly, and time management skills were more likely to be employed in NRI careers.

**Fig 2:**
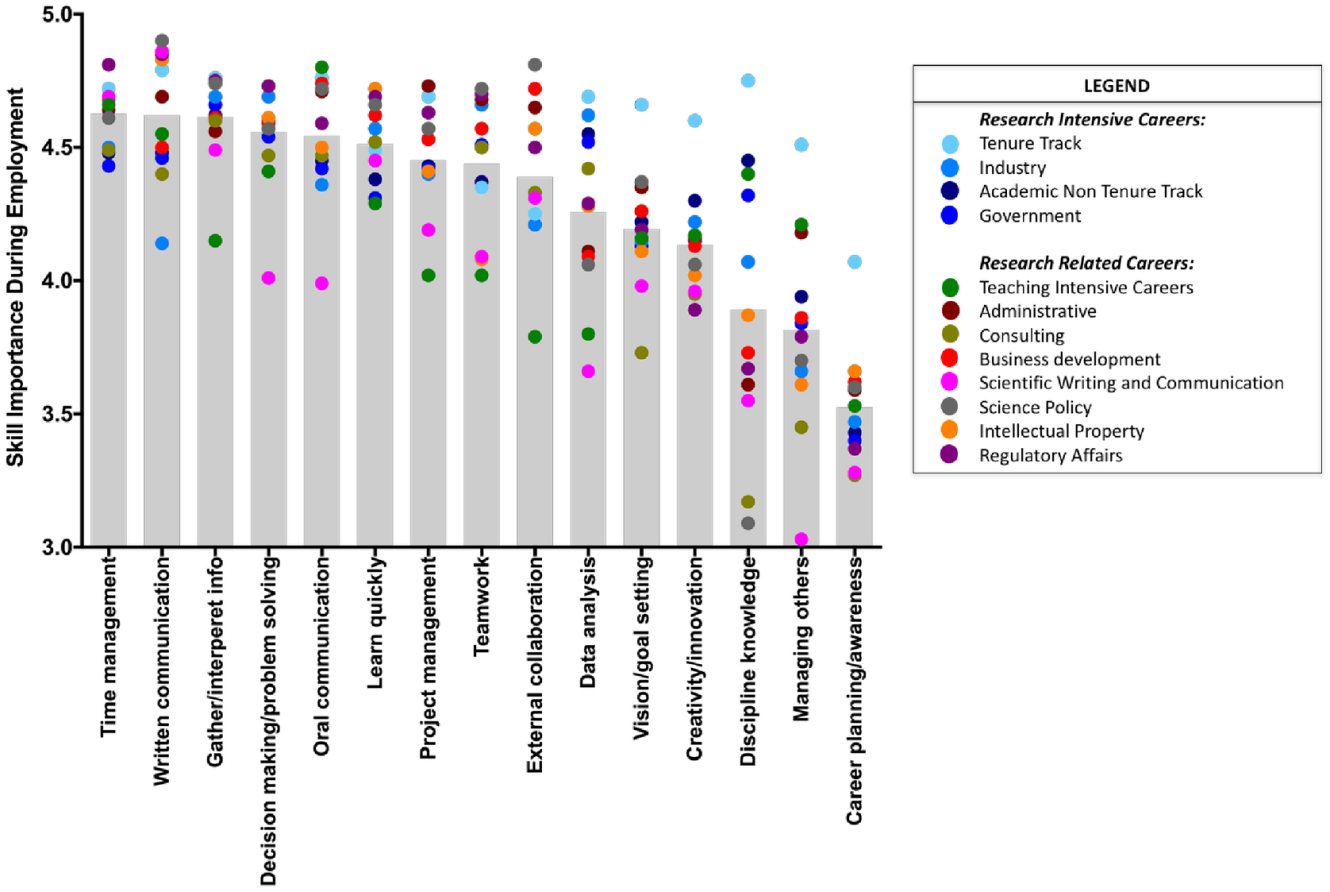
Transferrable skill importance for employment by career track. Currently employed PhDs rated the importance of each skill for their current role (“Other” responses not included on the plot above). The *overall mean importance rating* for each transferrable skill during employment is represented by a corresponding grey bar, ordered from left (highest) to right (lowest). Each *career track mean* is represented by a color-coded dot overlaid on the grey bar corresponding to each transferrable skill.

In addition to career differences in transferrable skills developed during doctoral training, we wanted to examine the importance of transferrable skills during employment. To examine transferrable skill distinctions at the more granular level of career tracks, we created a dot-plot (see Fig 2) of the importance of employment skills for four key RI careers, and eight common NRI research-related careers contained in the sample. Mean employment skill ratings of these twelve sub-categories were plotted as colored dots, on grey bars representing mean acquired doctoral skills. Two conclusions emerge: First, the importance of discipline-specific knowledge and ability to manage others varied the most widely across the careers. Second, while the majority of careers suggested the need for a broad skill set (e.g., tenure track, administrative, consulting), the data suggested that some careers may call for specialization in more narrow essential skill sets (e.g., science writing), although this should be interpreted with caution. It is also possible that the skills selected do not represent the totality of important skills pertinent to each career in question, with some key constructs left unassessed. Despite the small differences at this very granular level, the data points of most employment needs per skill set clustered fairly closely together reinforcing the conclusion that transferrable skills gained during PhD training prepare scientists broadly for many careers.

### Career choice and job satisfaction

Between the two major career categories for PhDs (RI and NRI), job satisfaction was relatively high, meaning that most respondents were satisfied or very satisfied in their current position (RI: 4.04 M, .99 SD; NRI: 4.08 M, 1.00 SD). We used an independent means *t*-test to examine whether there was a significant difference in job satisfaction between the two career groups. We found no significant difference in satisfaction between the two, *t*(3728) = 1.19, *p*>.234 (CI 95%: -0.03–0.10). This result suggests that scientists in NRI positions (*n* = 1741) were equally satisfied with their career compared with scientists in RI positions (*n* = 1989).

## Discussion

There are three overarching conclusions of this work. First, these results broadly suggest that doctoral programs provided trainees with a wide variety of transferrable skills. Second, the majority of those skills were transferrable across RI and NRI careers, with a few exceptions. Third, PhD-trained employees reported a high degree of job satisfaction, regardless of career choice. These findings are encouraging and lend support to ongoing efforts to broaden scientific training and career exploration opportunities during graduate education.

### Development of transferrable skills during doctoral training

#### Areas of strength

Our findings indicate that doctoral training programs in the sciences are providing trainees with transferrable skills that are valuable for a multitude of career paths. These skills include: discipline-specific knowledge, ability to gather and interpret information, ability to analyze data, oral communication skills, ability to make decisions and solve problems, written communication skills, ability to learn quickly, ability to manage a project, and creativity/innovative thinking.

#### Areas for improvement

There were, however, areas for which trainees ranked skill acquisition barely above neutral during their graduate training, indicating that there could be room to improve training in these areas during graduate school. The following skills were identified as less well-developed: the ability to set a vision and goals, time management, ability to work on a team, ability to collaborate outside the organization, ability to manage others, and career planning and awareness skills. Particularly of note, career planning and awareness ranked the lowest of all skills, suggesting an area for targeted growth in graduate education. Importantly, improvements in these skills are likely to be highly beneficial across a broad spectrum of both RI and NRI careers.

### Transferrable skills and career choices

The majority of skills we analyzed were not associated preferentially with either career category, suggesting that there is substantial overlap in the relevant skills needed for trainees who chose either RI or NRI careers.

#### Transferability of skills (RI and NRI)

Our study demonstrates that many transferrable skills are being developed in PhD training that are equally relevant across career paths. This finding suggests that doctoral programs in science fields are in many ways meeting the increasing demand to prepare graduates for a wide variety of careers. The majority of transferrable skills (66%) were similarly associated with both RI and NRI career choices. However, three skills were associated more with RI careers (career planning and awareness, creativity/innovation, and the ability to collaborate outside the organization) and three skills were associated more with NRI careers (project management, quick learning, and time management). Aside from these few exceptions, trainees who choose RI and NRI careers seem to share many of the same transferrable skills.

#### Research-intensive skills

Three skills were favorably associated with RI careers: creativity/innovation; career planning and awareness; and the ability to work with others outside the organization. The finding that creativity/innovation was positively associated with RI careers was not surprising. Both of these skills are crucial when developing new projects or using novel approaches, which are important aspects of a research intensive career.

Career planning and awareness shared a positive association with RI careers as well. One explanation for this association is that doctoral candidates interested in pursuing RI careers may be more likely to receive career guidance because their chosen career closely aligns with the training of their mentors, colleagues, and coworkers within the graduate training environment. Additional investigation could better examine the relationship between career planning with RI careers to determine what factors contribute to this association.

Ability to work with others outside the organization was also more highly associated with RI careers. Given the increased necessity for cross-disciplinary (and cross-institutional) scientific collaborations in academia and industry, this skill has become increasingly important and could explain the association with RI careers. It is worth noting that in addition to this skill being associated with an RI career, it also rises to the top of the skill gap list. Stated in different terms, finding ways to incorporate cross-sector collaborations into graduate training is likely to have significant positive outcomes for trainees on an RI career path.

As expected, postdoctoral experience was a significant predictor that associated positively with RI careers. This is likely because most RI careers expect independent research, demonstrated ability to attain grant funding, and a sustainable research program, which are central elements for a successful postdoctoral experience.

#### Non-research-intensive skills

Strengths in three skill categories were positively associated with NRI careers: project management; the ability to learn quickly; and time management. It is likely that individuals with research intensive graduate experiences who are transitioning to NRI careers need to adapt quickly to new settings, situations, and roles. This aspect of career transition could explain the positive association with the ability to learn quickly.

Likewise, the data suggest project management is a skill that is particularly well-developed by those who enter NRI careers. This skill is valued across a wide range of occupations because much work in the knowledge economy is project-based.

Another area for which higher skill ratings were associated with NRI careers was time management, which may be a part of the expectation for employees to complete work on time and by deadline in structured work environments. Time management is also fundamental to project-based work, signifying the importance of both of these skills to NRI careers.

#### Comparing skill acquisition during training and skill importance for employment

Skill importance during employment was compared with skill acquisition during doctoral training (e.g. Fig 1). Skill development during the PhD illustrated a similar pattern to the skill importance for employment when separated by broad career categories (RI and NRI): ratings of transferrable skills developed during graduate training were generally commensurate with the corresponding ratings of skill importance for employment. Further, skill importance ratings for most career types clustered near the means (e.g., Fig 2). These findings support the hypothesis that doctoral training programs in the sciences are generally preparing trainees well for the workforce across career pathways.

### Job satisfaction

PhDs who are employed in RI and NRI employment categories reported comparable levels of job satisfaction, challenging the prevailing notion that RI careers provide maximal career fulfillment for PhDs. Our data suggest that, for the most part, graduate training is preparing PhDs for both RI and NRI career fields, enabling them to be successful and satisfied in a wide variety of careers. This level of satisfaction is consistent with other recent national data sets (e.g., the 2013 Survey of Doctorate Recipients) which shows that scientists across the country and across occupations are largely satisfied with their work [9].

### Limitations

It is possible that some of the areas for potential improvement of graduate training identified in this study could simply represent skills where trainees lack confidence or awareness of their skill levels rather than an actual deficiency in skill-building opportunities during graduate school. Scientists who are accustomed to focusing on discipline-specific and technical expertise may be unaware that they have also acquired transferrable skills not directly related to their research pursuits. One potential solution to this lack of awareness would be to increase access to career counselors/coaches or other mentors who can help trainees identify the transferrable skills they already possess but may not recognize. For example, most scientists have collaborated with a range of colleagues including senior scientists (sometimes across departments or even institutions) and junior trainees (mentoring undergraduate research assistants, training new graduate students, etc.). These types of activities build communication, leadership, and teamwork skills. Many trainees may not recognize the degree of teamwork ability they have developed as part of a successful collaboration and fail to identify it as a personal strength until it is identified by another person. Similar reasoning may also apply to other acquired skills.

Furthermore, trainees may underestimate some skills they have acquired simply because additional improvements to the skill are desired. For example, no matter how proficient one becomes at time management, there is always room for improvement. This desire could result in a trainee’s perception of a time management deficiency that appeared as a skill gap in our analysis, when in fact, the trainee had indeed gained time management skills during their graduate training period. Nonetheless, it is still possible that more formal training in goal-setting and prioritization would improve time management skills for those engaged in graduate training in the sciences, as well as improving self-efficacy regarding time management.

Another limitation of our data is that we do not know the direction of causality between skill acquisition and career choice. We acknowledge that career choice represents an interplay between personal interests and skills, along with availability of jobs. Skills and techniques learned in a particular discipline may influence the feasibility of pursuing certain careers. Hence, it is possible that skills attained may encourage one to pursue a field where a particular skill is highly valued, or conversely that one’s career options may be limited if a highly valued skill is not a personal strength. In some cases, however, availability of jobs may strongly influence career choice. More research is needed to better understand the complex interaction of how skills, interests, and job availability interact to affect job choice.

Another methodological limitation of the sampling methods used is sample selection bias, since these methods largely assume online activity through the professional networking site LinkedIn, scientific societies, and other online networks. Furthermore, this sampling method may miss PhDs who are unemployed or not seeking employment and thereby less active on electronic or social media channels linked to this survey. Nonetheless, we believe these data are widely representative, inclusive of a large sample of PhDs from a variety of academic disciplines, and who hold a multitude of job types.

An additional concern, given the potential sampling bias, is that self-ratings of skill importance during employment may not match the actual skills employers are looking for. However, our employment skill rankings are quite similar to those collected from employer sources. The National Association of Colleges and Employers (NACE) Job Outlook 2014 survey reported similar means rated by employers for comparable transferrable skills [20]. This similarity suggests that the self-reported employee skill importance in our sample is representative of skills employers seek. While not all of the transferrable skills rated in the current sample overlapped with those in the 2014 NACE survey, those skills included in both samples showed similar patterns. This concurrence suggests that the skill gaps evident in our data set likely represent actual skill deficits in employees.

A related critique is that skills at higher levels on any organization chart may converge on managerial and leadership-relevant skills. However, our survey sample includes only those earning their PhDs within the ten-years prior to responding to the survey, thereby limiting the amount of career progression in the respondents. While this concern is a limitation (e.g., the current data are unable to address progression along a career path), the sample parameters nonetheless reduce the potential concern that career distinctions among fields could converge because senior PhDs may attain high-level leadership positions. In fact, this critique supports our conclusion that many skills are important to both RI and NRI career paths in that over time, those differences might diminish even further, again emphasizing the importance of most skills across career paths generally.

### Recommendations for graduate training programs

For those skills identified as possible areas for improvement in graduate training, our recommendation includes both expanding opportunities for trainees to gain awareness of skills developed as well as a greater programmatic focus on transferrable skill building. Therefore, we make two recommendations for graduate training programs: offer opportunities to a) improve trainee skill awareness, and b) enhance trainee skill acquisition.

First, improved skill awareness could be facilitated by an experienced career coach, counselor, or mentor who can help trainees to identify transferrable skills acquired during doctoral and postdoctoral training. Additionally, each trainee could learn to use terminology specific to the field to which they plan to apply. As a result, trainees will appropriately highlight the skills developed during their own training that are required for their chosen career. This process could also help graduates identify best fit careers which may lead to higher success in acquiring desired positions. Furthermore, recognizing their own abilities may improve trainees’ confidence in skills they have already developed (e.g., ability to learn quickly, ability to solve problems).

Second, increased resources for directed career training through an office responsible for graduate career and professional development is crucial for providing skill acquisition opportunities. An intentional focus on identifying and developing transferrable skills could be: 1) embedded into existing research experiences and program coursework, 2) developed by adding supplemental opportunities to existing training as *a la carte* programs, or 3) provided through one-on-one career counseling/coaching sessions with a professional. It is important to note that each institution may find specific combinations of these solutions most relevant and practical to implement. A recent initiative by the National Institutes of Health known as Broadening Experiences in Scientific Training (BEST) has a website [21] with resources and best practices that have emerged from 17 different institutions that have received BEST grants to develop new professional development programming.

In addition to improvements in these targeted skill growth areas for all trainees, institutions and programs may also consider providing specialized training experiences for those focused on RI careers (e.g., encouraging creativity/innovation) and for those targeting NRI careers (e.g., project management). We recognize that principal investigators are sometimes reluctant to support their trainees’ attendance at professional and career development events. However, the skills developed during training can benefit the trainee and their research projects immediately (e.g., communications, teamwork, problem-solving). Hence, administrators, faculty, and staff of doctoral training programs and related offices can be confident that building career and professional development training into the doctoral training period is a worthwhile investment and compatible with the overall doctoral training mission.

The outdated assumption that successful training for should exclusively prepare early career scientists for academic faculty positions has been challenged [17]. This expectation is a holdover from a time when a large proportion of junior scientists were expected to enter academic research positions, and when becoming a principal investigator was seen as the sole pinnacle of a science career. Successful transferrable skill development in conjunction with skill importance ratings by employees suggest that trainees are very competitive for and are highly successful in a multitude of careers. Given the intense competition for funding opportunities and limited positions available in the academic workforce compared to the number of trained scientists matriculating into the workforce, the apprentice-only model is particularly important to reexamine.

Our data on the connection between skill development and skill importance on the job, combined with the knowledge of comparable job satisfaction across careers, suggest that PhD programs should create, expand, or maintain professional development opportunities for trainees. Continued offerings that enhance career exploration and training for a broad range of RI and NRI careers should be considered as well. Trainees should be encouraged to pursue careers that align with their interests and skills, since we now know that rigorous training in scientific inquiry is vital for a range of occupations. Institutions and the scientific community should embrace this broader training model as central to their mission.

### Conclusions

These findings suggest that science PhDs enter the workforce equally well-prepared for both RI and NRI careers and remain equally happy in their chosen fields. Comparison of mean skill preparation with mean employee skill ratings suggests that doctoral training provides experiences that correspond to skill competency, as measured across all fifteen transferrable skills. Future directions of research could investigate discipline-specific differences in skill development—and the corresponding likelihood of entering RI vs. NRI careers—compared across academic fields of specialization. The conversation around broadening scientific career awareness, as well as increasing self-awareness among PhD graduates of their inherent skills, persists nationally and we hope for its continued evolution.

## Supporting information

S1 File Supporting data file (IBM SPSS file format). (SAV)

S2 File List of academic program survey options. (PDF)

S3 File Job title survey options. (PDF)
