# Supplementary material for: An evidence-based evaluation of transferrable skills and job satisfaction for science PhDs
Source: PLoS One. 2017 Sep 20;12(9):e0185023. doi: 10.1371/journal.pone.0185023 (PMC5607200; doi:10.1371/journal.pone.0185023)
Supplement: S2 File — (PDF) [file pone.0185023.s002.pdf]

S2 File. List of Academic Program Survey Options

In what field/discipline/academic program did you complete your doctorate?

- ❍ Accounting and Computer Science
- ❍ Acoustics
- ❍ Actuarial Science
- ❍ Aeronautical/Aerospace Engineering Technology/Technician
- ❍ Aeronautics/Aviation/Aerospace Science and Technology
- ❍ Aerospace Physiology and Medicine
- ❍ Aerospace, Aeronautical and Astronautical Engineering
- ❍ Aerospace, Aeronautical and Astronautical/Space Engineering
- ❍ Agribusiness/Agricultural Business Operations
- ❍ Agricultural and Domestic Animal Services
- ❍ Agricultural and Extension Education Services
- ❍ Agricultural and Food Products Processing
- ❍ Agricultural and Horticultural Plant Breeding
- ❍ Agricultural Animal Breeding
- ❍ Agricultural Business and Management
- ❍ Agricultural Business Technology
- ❍ Agricultural Communication/Journalism
- ❍ Agricultural Economics
- ❍ Agricultural Engineering
- ❍ Agricultural Mechanics and Equipment/Machine Technology
- ❍ Agricultural Mechanization
- ❍ Agricultural Power Machinery Operation
- ❍ Agricultural Production Operations
- ❍ Agricultural Public Services
- ❍ Agricultural/Farm Supplies Retailing and Wholesaling
- ❍ Agriculture, Agriculture Operations, and Related Sciences
- ❍ Agriculture, General
- ❍ Agroecology and Sustainable Agriculture
- ❍ Agronomy and Crop Science
- ❍ Air Transportation
- ❍ Algebra and Number Theory
- ❍ Analysis and Functional Analysis
- ❍ Analytical Chemistry
- ❍ Anatomy
- ❍ Animal Behavior and Ethology
- ❍ Animal Genetics
- ❍ Animal Health
- ❍ Animal Nutrition
- ❍ Animal Physiology
- ❍ Animal Sciences
- ❍ Animal Training
- ❍ Animal/Livestock Husbandry and Production
- ❍ Anthropology
- ❍ Applied Behavior Analysis
- ❍ Applied Economics
- ❍ Applied Horticulture and Horticultural Business Services
- ❍ Applied Mathematics
- ❍ Applied Psychology
- ❍ Aquaculture
- ❍ Aquatic Biology/Limnology
- ❍ Architectural and Building Sciences/Technology
- ❍ Architectural Drafting and Architectural CAD/CADD
- ❍ Architectural Engineering Technology/Technician
- ❍ Architectural Engineering
- ❍ Architectural History and Criticism
- ❍ Architectural Sciences and Technology
- ❍ Architectural Technology/Technician
- ❍ Architecture
- ❍ Artificial Intelligence
- ❍ Astronomy and Astrophysics
- ❍ Astronomy

- Astrophysics
- Atmospheric Chemistry and Climatology
- Atmospheric Physics and Dynamics
- Atmospheric Sciences and Meteorology
- Atomic/Molecular Physics
- Automation Engineer Technology/Technician
- Automotive Engineering Technology/Technician
- Aviation/Airway Management and Operations
- Behavioral Sciences
- Biochemical Engineering
- Biochemistry and Molecular Biology
- Biochemistry, Biophysics and Molecular Biology
- Biochemistry
- Bioengineering and Biomedical Engineering
- Bioethics/Medical Ethics
- Bioinformatics
- Biological and Biomedical Sciences
- Biological and Physical Sciences
- Biological/Biosystems Engineering
- Biology Teacher Education
- Biology, General
- Biology/Biological Sciences, General
- Biomathematics, Bioinformatics, and Computational Biology
- Biomedical Sciences, General
- Biomedical Technology/Technician
- Biomedical/Medical Engineering
- Biometry/Biometrics
- Biophysics
- Biopsychology
- Biostatistics
- Biotechnology
- Botany/Plant Biology
- CAD/CADD Drafting and/or Design Technology/Technician
- Cardiovascular Science
- Cell Biology and Anatomy
- Cell Physiology
- Cell/Cellular and Molecular Biology
- Cell/Cellular Biology and Anatomical Sciences
- Cell/Cellular Biology and Histology
- Ceramic Sciences and Engineering
- Chemical and Biomolecular Engineering
- Chemical Engineering Technology/Technician
- Chemical Engineering
- Chemical Physics
- Chemical Process Technology
- Chemical Technology/Technician
- Chemistry Teacher Education
- Chemistry
- City/Urban, Community and Regional Planning
- Civil Drafting and Civil Engineering CAD/CADD
- Civil Engineering Technology/Technician
- Civil Engineering
- Clinical Child Psychology
- Clinical Psychology
- Clinical, Counseling and Applied Psychology
- Cognitive Psychology and Psycholinguistics
- Cognitive Science
- Community Psychology
- Comparative Psychology
- Computational and Applied Mathematics
- Computational Biology
- Computational Mathematics
- Computational Science
- Computer and Information Sciences, General
- Computer and Information Systems Security/Information Assurance
- Computer Engineering Technology/Technician
- Computer Engineering
- Computer Graphics
- Computer Hardware Engineering

- Computer Hardware Technology/Technician
- Computer Programming
- Computer Science
- Computer Software and Media Applications
- Computer Software Engineering
- Computer Software Technology/Technician
- Computer Support Specialist
- Computer Systems Analysis
- Computer Systems Networking and Telecommunications
- Computer Teacher Education
- Computer Technology/Computer Systems Technology
- Computer/Information Technology Administration and Management
- Condensed Matter and Materials Physics
- Conservation Biology
- Construction Engineering Technologies
- Construction Engineering
- Corrections Administration
- Corrections and Criminal Justice
- Corrections
- Counseling Psychology
- Criminal Justice and Corrections
- Criminal Justice/Law Enforcement Administration
- Criminal Justice/Police Science
- Criminal Justice/Safety Studies
- Criminalistics and Criminal Science
- Crisis/Emergency/Disaster Management
- Critical Incident Response/Special Police Operations
- Critical Infrastructure Protection
- Crop Production
- Cultural Anthropology
- Cultural/Archaelogical Resources Protection
- Cyber/Computer Forensics and Counterterrorism
- Dairy Husbandry and Production
- Dairy Science
- Data Entry/Microcomputer Applications
- Data Modeling/Warehousing and Database Administration
- Data Processing and Data Processing Technology/Technician
- Data Processing
- Demography and Population Studies
- Development Economics and International Development
- Developmental and Child Psychology
- Developmental Biology and Embryology
- Drafting and Design Technology/Technician, General
- Drafting/Design Engineering Technologies/Technicians
- Earth Science Teacher Education
- Ecology and Evolutionary Biology
- Ecology, Evolution, Systematics, and Population Biology
- Ecology
- Econometrics and Quantitative Economics
- Economics
- Educational Psychology
- Electrical and Electronics Engineering
- Electrical Engineering Technologies/Technicians
- Electrical, Electronics and Communications Engineering
- Electrical/Electronics Drafting and Electrical/Electronics CAD/CADD
- Electromechanical Engineering
- Electromechanical Instrumentation and Maintenance Technologies/Technicians
- Electromechanical Technology/Electromechanical Engineering Technology
- Elementary Particle Physics
- Endocrinology

- Energy Management and Systems Technology/Technician
- Engineering Chemistry
- Engineering Design
- Engineering Mechanics
- Engineering Physics
- Engineering Physics/Applied Physics
- Engineering Science
- Engineering Technology, General
- Engineering-Related Fields
- Engineering-Related Technologies
- Engineering
- Engineering/Industrial Management
- Entomology
- Environmental Biology
- Environmental Chemistry
- Environmental Control Technologies/Technicians
- Environmental Design
- Environmental Design/Architecture
- Environmental Education
- Environmental Engineering Technology/Environmental Technology
- Environmental Health
- Environmental Psychology
- Environmental Science
- Environmental Studies
- Environmental Toxicology
- Environmental/Environmental Health Engineering
- Epidemiology
- Equestrian/Equine Studies
- Ethics
- Evolutionary Biology
- Exercise Physiology
- Experimental Psychology
- Family Psychology
- Farm/Farm and Ranch Management
- Financial Forensics and Fraud Investigation
- Financial Mathematics
- Fire Science/Fire-fighting
- Fire Services Administration
- Fire Systems Technology
- Fire/Arson Investigation and Prevention
- Fishing and Fisheries Sciences and Management
- Floriculture/Floristry Operations and Management
- Food Science and Technology
- Food Science
- Food Technology and Processing
- Forensic Chemistry
- Forensic Psychology
- Forensic Science and Technology
- Forest Engineering
- Forest Management/Forest Resources Management
- Forest Resources Production and Management
- Forest Sciences and Biology
- Forest Technology/Technician
- Forestry
- Game and Interactive Media Design
- Genetics
- Genome Sciences/Genomics
- Geochemistry and Petrology
- Geochemistry
- Geographic Information Science and Cartography
- Geography and Cartography
- Geography
- Geological and Earth Sciences/Geosciences
- Geological/Geophysical Engineering
- Geology/Earth Science, General
- Geometry/Geometric Analysis
- Geophysics and Seismology
- Geotechnical and Geoenvironmental Engineering
- Gerontology
- Geropsychology
- Greenhouse Operations and Management
- Hazardous Materials Information Systems Technology/Technician
- Hazardous Materials Management and Waste Technology/Technician
- Health Psychology

- Health/Medical Psychology
- Homeland Security
- Horse Husbandry/Equine Science and Management
- Horticultural Science
- Human Biology
- Human Computer Interaction
- Human/Medical Genetics
- Hydraulics and Fluid Power Technology/Technician
- Hydrology and Water Resources Science
- Immunology
- Industrial and Organizational Psychology
- Industrial Engineering
- Industrial Production Technologies/Technicians
- Industrial Radiologic Technology/Technician
- Industrial Safety Technology/Technician
- Industrial Technology/Technician
- Informatics
- Information Science/Studies
- Information Technology Project Management
- Information Technology
- Inorganic Chemistry
- Instrumentation Technology/Technician
- Integrated Circuit Design
- Interior Architecture
- International Agriculture
- International Economics
- Juvenile Corrections
- Kinesiology and Exercise Science
- Land Use Planning and Management/Development
- Landscape Architecture
- Landscaping and Groundskeeping
- Laser and Optical Engineering
- Laser and Optical Technology/Technician
- Law Enforcement Intelligence Analysis
- Law Enforcement Investigation and Interviewing
- Law Enforcement Record-Keeping and Evidence Management
- Law
- Library and Information Science
- Library Science
- Livestock Management
- Logic
- Management Sciences and Quantitative Methods
- Manufacturing Engineering Technology/Technician
- Manufacturing Engineering
- Marine Biology and Biological Oceanography
- Marine Sciences
- Maritime Law Enforcement
- Materials Chemistry
- Materials Engineering
- Materials Science
- Materials Sciences
- Mathematical Biology
- Mathematical Statistics and Probability
- Mathematics and Computer Science
- Mathematics and Statistics
- Mathematics Teacher Education
- Mathematics
- Mechanical Drafting and Mechanical Drafting CAD/CADD
- Mechanical Engineering
- Mechanical Engineering/Mechanical Technology/Technician
- Mechatronics, Robotics, and Automation Engineering
- Medical Anthropology
- Medical Illustration and Informatics
- Medical Illustration/Medical Illustrator
- Medical Informatics
- Medical Microbiology and Bacteriology
- Medicine
- Metallurgical Engineering

- Metallurgical Technology/Technician
- Meteorology
- Microbial and Eukaryotic Genetics
- Microbiological Sciences and Immunology
- Microbiology and Immunology
- Microbiology
- Military Science
- Mining and Mineral Engineering
- Mining and Petroleum Technologies/Technicians
- Mining Technology/Technician
- Modeling, Virtual Environments and Simulation
- Molecular Biochemistry
- Molecular Biology
- Molecular Biophysics
- Molecular Genetics
- Molecular Medicine
- Molecular Pharmacology
- Molecular Physiology
- Molecular Toxicology
- Museology/Museum Studies
- Museology/Museum Studies
- Mycology
- Nanotechnology
- Natural Resource Economics
- Natural Resource Recreation and Tourism
- Natural Resources and Conservation
- Natural Resources Conservation and Research
- Natural Resources Law Enforcement and Protective Services
- Natural Resources Management and Policy
- Natural Resources/Conservation, General
- Natural Sciences
- Naval Architecture and Marine Engineering
- Network and System Administration/Administrator
- Neuroanatomy
- Neurobiology and Anatomy
- Neurobiology and Behavior
- Neurobiology and Neurophysiology
- Neurobiology and Neurosciences
- Neuropharmacology
- Neuroscience
- Nuclear and Industrial Radiologic Technologies/Technicians
- Nuclear Engineering Technology/Technician
- Nuclear Engineering
- Nuclear Physics
- Nuclear/Nuclear Power Technology/Technician
- Nutrition Sciences
- Occupational Safety and Health Technology/Technician
- Ocean Engineering
- Oceanography, Chemical and Physical
- Oncology and Cancer Biology
- Operations Research
- Optics/Optical Sciences
- Organic Chemistry
- Ornamental Horticulture
- Other
- Packaging Science
- Paleontology
- Paper Science and Engineering
- Parasitology
- Pathology/Experimental Pathology
- Personality Psychology
- Petroleum Engineering
- Petroleum Technology/Technician
- Pharmaceutical Sciences
- Pharmacology and Toxicology
- Pharmacology
- Philosophy
- Photobiology
- Physical and Biological Anthropology
- Physical Chemistry
- Physical Science Technologies/Technicians
- Physical Sciences
- Physics Teacher Education
- Physics

- Physiological Psychology/Psychobiology
- Physiology
- Physiology, Pathology and Related Sciences
- Planetary Astronomy and Science
- Plant Genetics
- Plant Molecular Biology
- Plant Nursery Operations and Management
- Plant Pathology/Phytopathology
- Plant Physiology
- Plant Protection and Integrated Pest Management
- Plant Sciences
- Plasma and High-Temperature Physics
- Plastics and Polymer Engineering Technology/Technician
- Polymer Chemistry
- Polymer/Plastics Engineering
- Population Biology
- Poultry Science
- Psychology Teacher Education
- Psychology, Other
- Psychometrics and Quantitative Psychology
- Psychopharmacology
- Public Health, General
- Quality Control and Safety Technologies/Technicians
- Quality Control Technology/Technician
- Radiation Biology/Radiobiology
- Range Science and Management
- Real Estate Development
- Reproductive Biology
- Research and Experimental Psychology
- Robotics Technology/Technician
- School Psychology
- Science Teacher Education/General Science Teacher Education
- Science, Technology and Society
- Securities Services Administration/Management
- Security and Loss Prevention Services
- Semiconductor Manufacturing Technology
- Social Psychology
- Soil Chemistry and Physics
- Soil Microbiology
- Soil Science and Agronomy, General
- Soil Sciences
- Solar Energy Technology/Technician
- Statistics
- Structural Biology
- Structural Engineering
- Surveying Engineering
- Surveying Technology/Surveying
- Suspension and Debarment Investigation
- Sustainability Studies
- System, Networking, and LAN/WAN Management/Manager
- Systematic Biology/Biological Systematics
- Systems Engineering
- Systems Science and Theory
- Taxidermy/Taxidermist
- Telecommunications Engineering
- Telecommunications Technology/Technician
- Terrorism and Counterterrorism Operations
- Textile Sciences and Engineering
- Theoretical and Mathematical Physics
- Theoretical Chemistry
- Topology and Foundations
- Toxicology
- Transportation and Highway Engineering
- Turf and Turfgrass Management
- Urban Forestry
- Virology
- Vision Science/Physiological Optics
- Viticulture and Enology
- Water Quality and Wastewater Treatment Management and Recycling Technology/Technician

- Water Resources Engineering
- Water, Wetlands, and Marine Resources Management
- Web Page, Digital/Multimedia and Information Resources Design
- Web/Multimedia Management and Webmaster
- Welding Engineering Technology/Technician
- Wildland/Forest Firefighting and Investigation
- Wildlife and Wildlands Science and Management
- Wildlife Biology
- Wildlife, Fish and Wildlands Science and Management
- Wood Science and Wood Products/Pulp and Paper Technology
- Zoology/Animal Biology
