# Supplementary material for: An evidence-based evaluation of transferrable skills and job satisfaction for science PhDs
Source: PLoS One. 2017 Sep 20;12(9):e0185023. doi: 10.1371/journal.pone.0185023 (PMC5607200; doi:10.1371/journal.pone.0185023)
Supplement: S3 File — (PDF) [file pone.0185023.s003.pdf]

S3 File. Job Title Survey Options

"What is your current job title? If you hold a joint appointment, please select all that apply."

- ❑ Academic Advisor
- ❑ Adjunct Faculty
- ❑ Adjunct Instructor
- ❑ Analyst
- ❑ Artist
- ❑ Assistant Dean
- ❑ Assistant Director, Career Center
- ❑ Assistant Director, Postdoc Office
- ❑ Assistant Director, Scientific Programs
- ❑ Assistant Professor
- ❑ Associate Dean
- ❑ Associate Director, Career Center
- ❑ Associate Director, Postdoc Office
- ❑ Associate Director, Scientific Programs
- ❑ Associate Professor
- ❑ Astronomer
- ❑ Astrophysicist
- ❑ Biologist
- ❑ Biostatistician
- ❑ Botanist
- ❑ Business Development Analyst
- ❑ Career Counselor
- ❑ Chemist
- ❑ Chief Executive Officer
- ❑ Chief Scientific Officer
- ❑ Classroom Teacher
- ❑ Clinical Psychologist
- ❑ Clinical Trials Coordinator
- ❑ Clinical Trials Manager
- ❑ Consultant
- ❑ Curriculum Developer
- ❑ Data Analyst
- ❑ Data Scientist
- ❑ Dean
- ❑ Department Chair
- ❑ Director, Core Facility
- ❑ Director, Education
- ❑ Director, Postdoctoral Affairs
- ❑ Director, Research and Development
- ❑ Don't know
- ❑ Editor
- ❑ Educator, Scientific Society
- ❑ Engineer
- ❑ Entrepreneur
- ❑ Epidemiologist
- ❑ Field Application Specialist
- ❑ Genetics Counselor
- ❑ Grants Administrator
- ❑ Graphic Designer
- ❑ Group Leader
- ❑ Imaging Specialist
- ❑ Instructor
- ❑ Investment Analyst
- ❑ Journalist
- ❑ Laboratory Manager
- ❑ Lecturer
- ❑ Management Consultant
- ❑ Manager
- ❑ Market Researcher
- ❑ Marketing Specialist
- ❑ Mathematician
- ❑ Medical Informaticist
- ❑ Medical Science Liaison
- ❑ Medical Writer
- ❑ Museum Educator
- ❑ Neuroscientist
- ❑ Other
- ❑ Patent Agent
- ❑ Patent Attorney
- ❑ Patent Law Clerk
- ❑ Physician
- ❑ Physicist
- ❑ Policy Analyst
- ❑ President
- ❑ Principal Investigator
- ❑ Product Development Scientist/Engineer
- ❑ Professor
- ❑ Program Analyst
- ❑ Program Manager

- ❑ Program Officer
- ❑ Project Manager
- ❑ Public Outreach Specialist
- ❑ Quality Control Specialist
- ❑ Quantitative Researcher
- ❑ Regulatory Affairs Specialist
- ❑ Research Administrator
- ❑ Research Analyst
- ❑ Research Assistant
- ❑ Research Associate
- ❑ Research Assistant Professor
- ❑ Research Associate Professor
- ❑ Research Professor
- ❑ Research Scientist
- ❑ Researcher
- ❑ Science Writer
- ❑ Scientist
- ❑ Senior Lecturer
- ❑ Senior Quantitative Researcher
- ❑ Senior Scientist
- ❑ Senior Staff Scientist
- ❑ Staff Scientist
- ❑ Statistician
- ❑ Technical Sales Representative
- ❑ Technical Support Specialist
- ❑ Technical Writer
- ❑ Technology Transfer Specialist
- ❑ Therapist
- ❑ Venture Capital Analyst
- ❑ Vice President, Research and Development
- ❑ Visiting Assistant Professor
- ❑ Visiting Associate Professor
- ❑ Visiting Professor
